# Cluster Migration Distance for Performance Degradation Assessment of Water Pump Bearings

**DOI:** 10.3390/s22186809

**Published:** 2022-09-08

**Authors:** Zhongping Zhai, Zihao Zhu, Yifan Xu, Xinhang Zhao, Fang Liu, Zhihua Feng

**Affiliations:** 1 Department of Precision Mechanics and Precision Instruments, University of Science and Technology of China, Hefei 230027, China; 2 School of Electrical Engineering and Automation, Anhui University, Hefei 230601, China

**Keywords:** water pump bearing, fault diagnosis, blind source separation, feature fusion, feature distance, dynamic center of mass

## Abstract

Because the signal of water pump bearing is seriously disturbed by noise and the fault evolution is complex, it is difficult to describe the performance degradation trend of water pump bearing in a timely and accurate manner using the traditional performance degradation index (PDI). In this paper, a new Cluster Migration Distance (CMD) algorithm is proposed. The extraction of the indicator includes the following four steps: First, the relevant blind separation is used to extract the useful signal of the monitored bearing from the mixed signal; secondly, the impact component is further enhanced by wavelet packet analysis. Then, the redundancy of the original feature vectors is eliminated using our previously proposed KJADE (Kernel Joint Approximate Diagonalization of Eigen-matrices) method. Finally, the newly proposed CMD index is computed as PDI. By calculating the offset trajectory of the feature cluster centroid in the continuous running process of the bearing, CMD can aptly deal with the complex and variable features in the fault evolution process of the water pump bearing. The whole-life monitoring data of a 220 KW water pump system are processed. The results show that the proposed CMD index has better early-warning ability and monotonicity than the traditional kurtosis index.

## 1. Introduction

Water pumps play an important role in urban drinking water treatment, agricultural irrigation, and other scenarios. As one of the key components of water pumps, bearings are of great significance to ensure their healthy and reliable operation. However, due to their high load and high-speed operation over a long duration, various kinds of faults inevitably occur [1,2,3].

Dutta N. et al. [4]. proposed an artificial neural network-based model for the diagnosis of water pump bearings with higher accuracy than other machine-learning algorithms. Tang Jing et al. [5] converted the collected vibration signals into frequency domain signals and used frequency domain features and support vector machines to diagnose the faults of water pump bearings. Li Tao et al. [6,7] proposed an adaptive algorithm based on a particle swarm optimization algorithm using the convolutional neural network fault diagnosis method. Other research [8] proposed the method of the wavelet packet to denoise the vibration signal of the centrifugal pump bearing and extracted the frequency band energy representing the corresponding bearing fault and used a BP neural network for training and fault identification. Han Hui et al. [9] proposed a water pump bearing diagnosis method based on stack noise reduction and self-encoding, which obtained a more robust feature representation and could precisely diagnose the fault state of water pump bearings. Bie Zhongzheng et al. [10] introduced the principle of peak energy analysis and used the peak energy value to judge the failure degree of the water pump bearing.

Denoising and accurate performance degradation index extraction are two major challenges in achieving effective performance degradation assessment of water pump bearings. In practical engineering applications, the water pump is usually driven to rotate by a motor through a coupling, and four bearings are usually installed coaxially on the main shaft. Therefore, the vibration signal of each bearing will receive vibration interference from other bearings, which introduces difficulties in the accurate extraction of the weak fault information in the early stages of the bearing. Therefore, it is necessary to study effective means to effectively eliminate noise. Bearing failures, on the other hand, have complex evolutionary processes, often starting with one failure type, such as localized material tribes, and then more complex composite failures may emerge as bearing components interact with localized defects. Therefore, accurately characterizing the performance degradation trend of water pump bearings is one of the main challenges.

Regarding research on noise reduction, Pinlu et al., proposed a deep learning method for efficient noise reduction and feature extraction, which is based on a combination of residual construction units, soft thresholding, and global context. However, this method has the disadvantage of a shared threshold [11]. Sandaram Buchaiah and Piyush Shakya et al., applied different signal processing techniques to extract 72 raw features from vibration data collected experimentally on bearings. They used a random forest method to select a subset of relevant features from the extracted features. The selected features were fused through a 14-dimensional dimensionality-reduction technique, from which two-dimensional relevant performance indicators are extracted and compared between techniques to determine the most effective technique. However, these algorithms are numerous and they need to be compared when they are used, and it is impossible to determine which one is the optimal algorithm [12]. Fei-Ping Du et al., proposed an adaptive regularization parameter selection strategy to denoise vibration signals using a sparse redundant representation model. However, this posterior method requires a great deal of computation of the FP metric, and although this method is very effective, it has not been applied to all scenarios [13].

Regarding research on performance degradation indicators, Yang Chuang Yan et al., established a new RUL prediction model for the problems of poor residual life prediction performance and the single performance degradation index of rolling bearings, and the method showed a good performance in prediction accuracy and reliability [14]. Yan Xiao li et al., proposed a bearing performance degradation evaluation algorithm based on CMMP and feature fusion. Mathematical morphological operations are driven by partial differential equations (PDEs) for the accurate assessment of bearing life cycle failure datasets [15]. Tao Zan et al., introduced joint approximate diagonalization of a characteristic matrix (JADE) and a particle swarm optimization support vector machine (PSO–SVM) in the prediction of the performance degradation trend of rolling bearings, and realized the performance degradation trend and residual performance of rolling bearings under small samples, with an accurate prediction of service life [16].

In the above studies, it is generally considered that only one type of failure occurs in the bearing during the degradation process, but in fact, in actual engineering cases, we often find that there are composite failures. This is because, after the early failure of a bearing, the interaction of the local failure with the bearing components can lead to more complex compound failures. At this time, some traditional performance degradation indicators, such as kurtosis, will fluctuate significantly, which affects the accurate judgment of the performance degradation trend. Therefore, in this study, we innovatively propose a novel performance degradation metric extraction method called Cluster Migration Distance (CMD). CMD does not focus on the growth of features, but rather on the change in features, becoming lower or higher. Degradation trends are assessed by tracking changes in features. At the same time, in order to solve the problem of strong noise interference, we comprehensively use the non-target vibration source interference elimination ability of blind separation and the impact component identification ability of wavelet analysis to achieve noise elimination and use our specially proposed KJADE method for feature fusion processing. In order to verify the effectiveness of the proposed method, we verify the life-cycle monitoring data of a 220 KW water pump system in practical engineering applications. The results show that the early warning ability and the monotonicity of the proposed CMD index are better than the traditional kurtosis indicators.

The details of this work are described in the following sections. Section 2 provides the detailed flow of the proposed approach. Section 3 presents the analysis results and related discussions of an engineering application case. Section 4 concludes this work and points out future research work.

## 2. Method and Process

In practical engineering applications, the early fault signal of a water pump bearing is seriously interfered with, and the fault evolution process is complex and changeable. For the purpose of solving this problem, this paper proposes a new performance degradation index extraction method called the Cluster Migration Distance (CMD). At the same time, blind separation, wavelet packet analysis, and the KJADE feature fusion method are comprehensively used to assist in the accurate extraction of bearing performance degradation indicators.

The proposed method mainly includes the following four steps: (1) In order to eliminate the interference of the coaxial vibration source in the pump system, decorrelated blind separation is used to separate the signal of the monitored bearing from the acquired signals of multiple measurement points. (2) Wavelet packet analysis is used to further enhance the shock components closely related to the diagnostic information from the signal; the comprehensive application of the blind separation and wavelet packet analysis methods can effectively eliminate noise interference. (3) Our proposed KJADE method is used to eliminate redundancy and feature fusion on the original time–frequency domain feature vectors; the KJADE method combines the nonlinear processing capability of the kernel method and the advantages of high-order cumulant calculation in the JADE method, which can effectively extract features that characterize fault information. (4) Lastly, the CMD index is calculated to be used as a final indicator to describe the degradation trend of the monitored bearing performance. 

**Figure 1:**
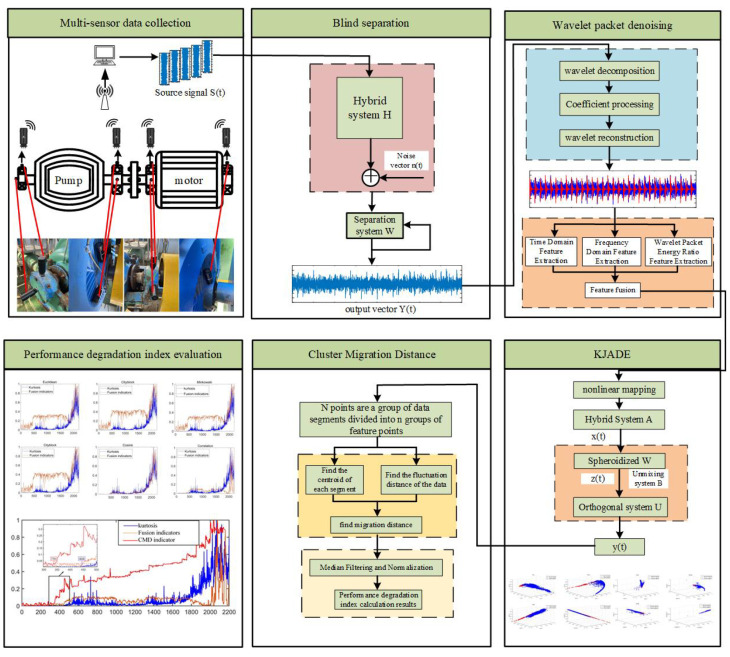
Overall flow chart.

**Figure 2:**
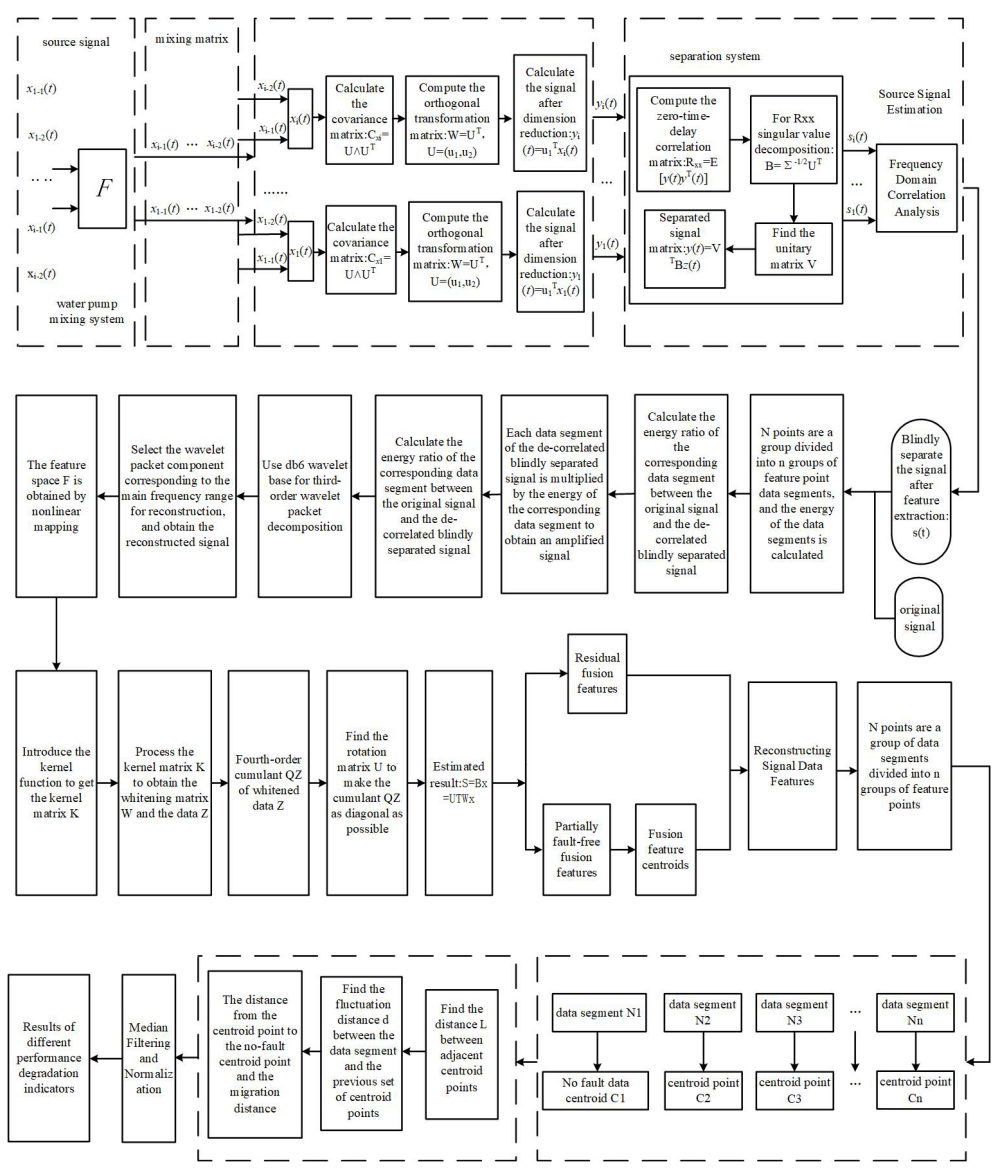
Overall algorithm flow chart.

The schematic flow chart of the method and the detailed flow chart of the algorithm are shown in Figure 1 and Figure 2, respectively, and the details of the method will be described below.

### 2.1. Vibration Source Interference Cancellation Based on Blind Separation

Principal Component Analysis (PCA) [13,16] is a multidimensional data analysis method commonly used in statistical analysis, and it is able to find implicit statistical features from raw data. It is very effective in data dimensionality reduction [17], information compression, and de-correlation between data. In this section, PCA is used to reduce the dimensionality of the two-dimensional signals collected by the horizontal and vertical sensors of the same measuring point before blind separation and extract the main feature information. We assume that the vibration source signal of the monitored bearing is *s*(*t*), and other coaxial vibration source signals are *s*_i_(*t*). After the pump mixing system, the random vector formed by the observation signals of the same measuring point is $\;x={\left(x_{i-1},x_{i-2}\right)}^{T}$, (i = 1,…, *N*), *N* is the number of measuring points, and its mean $m_{x}=E\left(x\right)=0$. We then find an orthogonal transformation matrix ***W*** and perform an orthogonal transformation on the random vector, so that the random variables in the output random vector *y* = ***W****x* are not correlated with each other.

(1) $$C_{y}=E\left\{yy^{T}\right\}=diag\left(\lambda_{1},\lambda_{2}\right)$$

The orthogonal matrix W is obtained by decomposing the eigenvalues of the covariance matrix ***C****_x_* of x. Usually, ***C****_x_* is a real symmetric matrix, decomposed as:

(2) $$C_{x}=U\Lambda U^{T}$$

where $U=\left(u_{1},u_{2}\right)$, $\Lambda =diag\left(\lambda_{1},\lambda_{2}\right)$ and $u_{1}$ and $u_{2}$ are the eigenvectors of the covariance matrix ***C****_x_*. The eigenvectors are orthogonal to each other, that is $E\left\{u_{i}{}^{T}u_{j}\right\}=0\left(i\neq j\in 1,2\right)$, and $\lambda_{i}$ is the corresponding eigenvalue and is presented as:

(3) $$\lambda_{i}=E\left\{{\left(u_{i}{}^{T}x\right)}^{2}\right\}>\lambda_{j}=E\left\{{\left(u_{j}{}^{T}x\right)}^{2}\right\},i<j\in 1,2$$

It can be seen that PCA establishes a set of orthogonal bases $u_{1},u_{2}$ for the 2-dimensional data space, and $u_{1}\;\mathrm{is}\;\mathrm{orthogonal}\;\mathrm{to}\;u_{2}$.$y_{i}=u_{i}{}^{T}x,i=1,2$. We then take the first *y_i_*(*t*) after dimensionality reduction as the principal component.

#### Blind Separation of Decorrelation

In the previous link, the dimensionality reduction in the two-dimensional signals is measured by the horizontal and vertical displacement sensors of the same vibration measuring point. The signal matrix ***y***(*t*) formed by each measuring point after dimension reduction is used as the observed signal matrix for de-correlated blind separation, and feature extraction is achieved using de-correlated blind separation [18,19,20]. Assuming that the signals have statistical irrelevance, non-whiteness, or non-stationarity, and there is no more than one Gaussian signal in the signal, the eigenvalue decomposition of multiple non-zero time-delay correlation matrices is used to achieve blind signal separation. We then proceed as follows:

(1) The observed signal is pre-whitened. We calculate the zero-time delay correlation matrix *R_xx_* of the observed signal matrix ***y***(*t*) and perform singular value decomposition,

(4) $$R_{xx}=E\left[y\left(t\right)y^{T}\left(t\right)\right]=U\sum U^{T},B=\sum^{-1/2}U^{T}$$

where Σ is a diagonal matrix consisting of singular values and then $z\left(t\right)=By\left(t\right)$.

(2) We find unitary matrices *V* and make the set of non-zero time-delay correlation matrices $\left\{R_{zz}\left(\tau_{1}\right),R_{zz}\left(\tau_{2}\right),\cdots ,R_{zz}\left(\tau_{k}\right)\right\}$ joint diagonalization. The objective function is as follows:

(5) $$f\left(V\right)=\sum_{k=1}^{k}off\left(V^{H}R_{zz}\left(\tau_{k}\right)V\right)$$

Among them, *k* is the number of time delay correlation matrices, $off\left(M\right)=\sum_{1<i\neq j<n}{\left|M_{ij}\right|}^{2}$.

(3) The separated signal matrix is:

(6) $$s\left(t\right)=V^{T}z\left(t\right)$$

The frequency domain correlation analysis is carried out between the separated signal and the monitored bearing signal, and the separated signal with a high-frequency domain correlation is taken as the monitoring bearing signal after blind separation.

### 2.2. Wavelet Packet Analysis to Achieve Shock Component Enhancement

**Figure 3:**
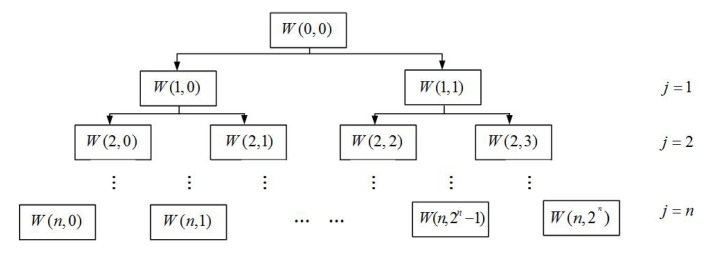
Wavelet decomposition tree.

After blind separation, the signal *s*(*t*) is subjected to wavelet packet decomposition, and its decomposition tree is shown in Figure 3.

If the sampling frequency of the bearing vibration signal is *f_s_*, after the n-layer wavelet packet analysis, there are a total of 2*^n^* nodes, and the frequency interval of each node segment is $\frac{fs}{2^{n+1}}$.

By analyzing the spectrum of the signal *s*(*t*), the concentration area of the fault’s frequency components is determined, and then the corresponding node is selected for reconstruction to obtain the signal *x*(*t*).

### 2.3. Multivariate Feature Extraction in Time–Frequency Domain

After the vibration sensor collects the bearing signal, extracting the features reflecting the bearing state from these data is a key step in realizing fault diagnosis. The quality of the extracted features directly affects the recognition accuracy. Due to the complexity of the bearing structure and the superposition and coupling of the vibration signals of various components, a single characteristic index, such as the root mean square value or peak value, cannot accurately reflect the current state of the bearing [21]. It can, however, comprehensively evaluate the running state of the bearing [22]. Therefore, the time-domain dimensionless index, the frequency-domain index, and the energy ratio index based on the wavelet packet sub-band are extracted as the original high-dimensional feature vectors.

#### 2.3.1. Extraction Method of Time-Domain Features

The signal obtained by wavelet packet decomposition and reconstruction is *x*(*t*), and *n* is the number of sampling points. The extracted signal feature set has dimensional indicators and non-dimensional indicators [23], as shown in Table 1.

#### 2.3.2. Extraction Method of Frequency Domain Features

When the bearing is in a healthy state, its vibration and acoustic signals are small during operation. When the bearing is damaged locally, a periodic impact signal will be generated, which will lead to the high-frequency vibration of the bearing itself. Therefore, the vibration signal of the bearing is very complex, and different types of bearing faults have different fault frequencies and impact laws. Therefore, the complex time domain signal can be transformed into a single harmonic component through Fourier transform for research, so as to obtain each harmonic component, such as the amplitude and phase information of the wave. Since the signals of different fault types do not have exactly the same frequency spectra, different frequency domain characteristic parameters are required for monitoring [24]. F13–F16, as listed in Table 1, are the frequency domain features required in this chapter, where *f_i_* and *s_i_* are the frequency and amplitude corresponding to the *i*-th spectral line of the reconstructed signal *x*(*t*).

#### 2.3.3. Extraction Method of Time–Frequency Domain Features

As a typical time–frequency domain analysis method, wavelet decomposition can perform multi-scale transformation of vibration signals. Since the fault information widely exists in different frequency components in the signal, a change in the frequency component often indicates that the state of the bearing has changed, so wavelet packet frequency band energy detection technology can be used to realize the original feature extraction of the bearing’s operating state.

Third-order wavelet packet decomposition is performed on the reconstructed signal *x*(*t*), and the energy corresponding to the node *x*_3*i*_ is *E_i_*, while the corresponding discrete point amplitude is *h_ik_*, i=0,1,2,…,7;k=1,2,…,n, and *n* is the number of sampling points. Then, the energy of the i-th subband signal is:

(7) $$E_{i}=\int {\left|x_{3i}\right|}^{2}dt=\sum_{k=1}^{n}{\left|h_{ik}\right|}^{2}$$

In order to more intuitively judge the change in energy and perform dimensionless processing on the extracted sub-band energy of wavelet packets, the energy ratio of each frequency band energy to the total energy *E*, as shown in Table 1, can be obtained as the time–frequency domain energy ratio original features, of which the total energy $E=\sum_{i=0}^{7}E_{i}$.

### 2.4. Advanced Feature Fusion Extraction Based on KJADE

**Figure 4:**
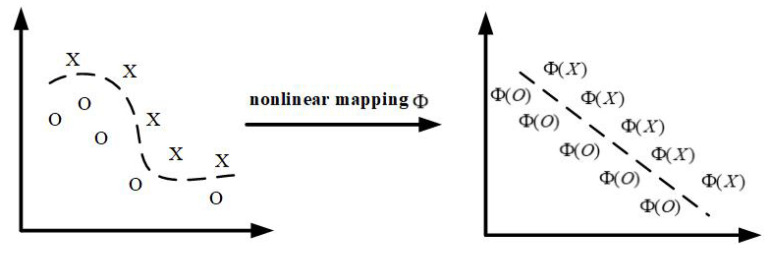
Nonlinear mapping.

In the previous section, time-domain indicators, frequency-domain indicators, and energy ratios based on wavelet packet sub-bands were extracted from the vibration signal, and formed a multi-domain feature set, avoiding the shortcoming of the insufficient evaluation capability of a single feature. However, there are redundant and conflicting problems among some features in multi-domain features, so it is necessary to extract feature quantities sensitive to bearing fault states. The bearing vibration signal often has nonlinear characteristics, and JADE belongs to the linear processing method, so it cannot effectively extract the characteristic quantities sensitive to the bearing fault state. In order to further apply JADE to bearing vibration signals with nonlinear behavior, the idea of the kernel function method is introduced to JADE, and the joint approximate diagonalization based on the kernel function eigenmatrix is obtained. Kernel Feature Matrix Joint Approximate Diagonalization (KJADE) is a novel feature fusion method. It not only has the characteristics of JADE, but also has better nonlinear processing capabilities than JADE. The core idea is to map the data $X\in R^{n\times m}$ to the high-dimensional feature space F through the nonlinear function Φ, and then use the JADE algorithm to transform the nonlinear separable problem into a linearly separable problem in *F*. The mapping process is shown in Figure 4. Assuming that the sample space is $X=\left\{x_{1},x_{2},\ldots ,x_{n}\right\}$, where *x_i_* is the input vector of the *i*-th dimension of the sample space, which contains *n* data points, after mapping, the feature space is $F=\left\{\Phi \left(x_{1}\right),\Phi \left(x_{2}\right),\ldots ,\Phi \left(x_{M}\right)\right\}$.

For the same purpose as JADE, KJADE also needs to find unmixed matrix B to obtain the optimal matrix, namely:

(8) $$y=B^{F}\Phi \left(x\right)=U^{T}W\Phi \left(x\right)$$

Furthermore, the covariance matrix of the mapped *F* can be obtained as:

(9) $$R_{F}=\frac{1}{N}\Phi \left(x_{i}\right)\Phi {\left(x_{i}\right)}^{T}=\frac{1}{N}FF^{T}$$

*R_F_* and its eigenvalues and eigenvectors cannot be obtained due to the “curse of dimensionality” caused by the excessive dimension of the feature space. The idea of kernel function is introduced here, and the complex and time-consuming inner product calculation is converted into a kernel function, and an *N* × *N* kernel matrix *K* is obtained:

(10) $$K_{ij}=\Phi \left(x_{i}\right)\Phi \left(x_{j}\right)=k\left(x_{i},x_{j}\right)$$

Among them, *K_ij_* must meet the Mercer condition, that is, *K* = *FF^T^*. The following are commonly used kernel functions:

Gaussian kernel function:

(11) $$k\left(x_{i},x_{j}\right)=\exp(-\frac{x_{i}-x_{j}{}^{2}}{2\sigma^{2}})$$

Polynomial Kernel Function:

(12) $$k\left(x_{i},x_{j}\right)={\left(ax_{i}^{T}x_{j}+c\right)}^{d}$$

Sigmoid Kernel function:

(13) $$k\left(x_{i},x_{j}\right)=\tan h(ax_{i}^{T}x_{j}+c)$$

Since the Gaussian kernel function achieves better results in solving practical problems [25], the Gaussian kernel function is used in this study to replace the inner product operation, where σ represents the width parameter of the function. Then, the eigendecomposition of the fourth-order cumulant matrix of the extracted kernel matrix is produced, and the nonlinear feature fea_kjade(*x_i_*,*y_i_*,*z_i_*) hidden in the observation signal is obtained.

### 2.5. Calculation of CMD Performance Degradation Index

When the bearing fails, it has good class separability from the characteristic distribution of the healthy bearing. With the continuous operation of the bearing, the failure degree is expanded, and the characteristic difference between the monitored bearing and the healthy bearing becomes larger. Therefore, two types of models can be constructed to evaluate the difference between monitoring signals and health signals. In the classification of samples, the intra-class distance and the inter-class distance have been applied in the class separability measurement between different bearing fault types.

**Figure 5:**
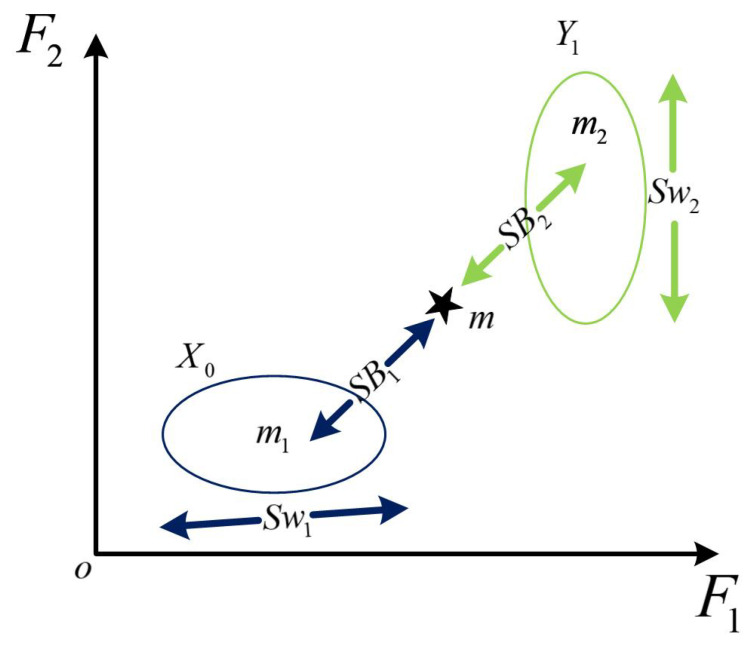
Schematic diagram of the two types of models.

The two types of models constructed are shown in Figure 5. In the extracted nonlinear feature fea_kjade(*x_i_*,*y_i_*,*z_i_*), we extract the feature segment of the bearing in normal operation and record the feature set extracted in the healthy state of the bearing as $X_{o}$, the feature set extracted by the bearing at time t is $Y_{t}$, and then the two types of models formed are $\;Z_{t}=\left[X_{o},Y_{t}\right]$, where $X_{o},Y_{t}=\left(x_{1},x_{2},\ldots ,x_{i},\ldots ,x_{n}\right)$, *n* is the number of samples, $x_{i}\in R^{D}$ (D is the feature dimension). Then, the inter-class scatter matrix is:

(14) $$S_{b}=\sum_{i=1}^{c}P_{i}m_{i}-m^{2}$$

The intra-class scatter matrix is:

(15) $$S_{w}=\sum_{i=1}^{c}P_{i}\frac{1}{n_{i}}\sum_{k=1}^{n_{i}}x_{k}^{i}-m_{i}{}^{2}$$

In:

(16) $$\left\{\begin{array}{c} P_{1}=n_{i}/\sum_{j=1}^{c}n_{j}\\ m_{i}=1/n_{i}\sum_{k=1}^{n_{i}}x_{k}^{i}\\ m=\sum_{i=1}^{c}p_{i}m_{i} \end{array}\right.\;\;\;\;\;1\leq i,j\leq C$$

*C* is the number of classes, where *C* = 2, and *m_i_* and *m* are the feature mean of class *i* and the entire sample, respectively. The inter-class scatter matrices *S_b_* and *S_w_* represent the degree of aggregation between different classes and between the same class, respectively. To describe the feature part more comprehensively, the intra-class and inter-class distance *SS* is used as a measure of class separability, and its expression is shown in Equation (17).

(17) $$SS=trace\left(S_{b}/S_{w}\right).$$

### 2.6. Performance Degradation Indicator

#### 2.6.1. Selection of Distance Index Calculation

Because clustering does not know any sample labels, the samples are divided into different classes through the internal relationship and different calculation methods are used, and the clustering will also obtain different results. The following are commonly used similarity calculation methods. Therefore, when calculating small sample clustering, it is necessary to calculate the relevant distance. When calculating the distance between the feature set $Y_{t}$ extracted at time t and the centroid of the feature set X0 extracted under the healthy sample, we use the following distance formulas as a reference for comparison:

1. Euclidean Distance

According to the calculation, this distance can be considered to be the L2 norm. Two points a(*x*_1_,*x*_2_…*x_n_*) and b(*y*_1_,*y*_2_…*y_n_*) are in the n-dimensional space:

(18) $$d_{ab}=\sqrt{{\left(x_{1}-y_{1}\right)}^{2}+{\left(x_{2}-y_{2}\right)}^{2}+\cdots +{\left(x_{n}-y_{n}\right)}^{2}}$$

2. Manhattan Distance

According to the calculation, this distance can be considered to be the L1 norm. Two points a(*x*_1_,*x*_2_…*x_n_*) and b(*y*_1_,*y*_2_…*y_n_*) are in the n-dimensional space:

(19) $$d_{ab}=\left|x_{1}-y_{1}\right|+\left|x_{2}-y_{2}\right|+\cdots +\left|x_{n}-y_{n}\right|$$

3. Chebyshev Distance

It is simply considered the maximum value of the coordinate difference of each coordinate. Two points a(*x*_1_,*x*_2_…*x_n_*) and b(*y*_1_,*y*_2_…*y_n_*) are in the n-dimensional space:

(20) $$d_{ab}=max\left(\left|x_{1}-y_{1}\right|,\left|x_{2}-y_{2}\right|,\ldots \left|x_{n}-y_{n}\right|\right)$$

4. Minkowski Distance

The Minkowski distance between two n-dimensional variables a(*x*_11_,*x*_12_,…,*x*_1*n*_) and b(*x*_21_,*x*_22_,…,*x*_2*n*_) is defined as:

(21) $$d_{12}=\sqrt[{p}]{\sum_{k=1}^{n}{\left|x_{lk}-x_{2k}\right|}^{p}}$$

where p is a variable parameter. Min’s distance defines a set of distance formulas, including the Euclidean distance, Manhattan distance, and Chebyshev distance.

5. Cosine Distance

The cosine similarity derivation formula is as follows:

(22) $$\cos\left(\theta \right)=\frac{a^{2}+b^{2}-c^{2}}{2ab}$$

Two points a(*x*_1_,*x*_2_…*x_n_*) and b(*y*_1_,*y*_2_…*y_n_*) are in the n-dimensional space

(23) $$\cos(\theta )=\frac{\sum_{k=1}^{n}x_{1k}x_{2k}}{\sqrt{\sum_{k=1}^{n}x_{1k}{}^{2}}\sqrt{\sum_{k=1}^{n}x_{2k}{}^{2}}}$$

6. Correlation Distance

The correlation coefficient is a way to assess the degree of correlation between random variables *X* and *Y*.

(24) $$\rho_{\mathrm{XY}}=\frac{Cov\left(X,Y\right)}{\sqrt{D\left(X\right)}\sqrt{D\left(Y\right)}}=\frac{E(\left(X-EX\right)\left(Y-EY\right)}{\sqrt{D\left(X\right)}\sqrt{D\left(Y\right)}}$$

Correlation distance:

(25) $$Dxy=1-\rho_{XY}$$

#### 2.6.2. Dynamic Centroid

When the bearing is running, its characteristic signal data migrate and change. In order to more clearly observe the migration and change trend of the fusion features, we propose the concept of the dynamic centroid. When finding the centroid of a single data cluster, it is often best to find a fixed number of K centroid points for the entire data cluster and iteratively find a partition scheme for K clusters, minimizing the loss corresponding to the clustering result function, where the loss function can be defined as the sum of squared errors between the sample points in each cluster and their center points:

(26) $$J\left(c,\mu \right)=\sum_{i=1}^{M}x_{i}-\mu_{ci}{}^{2}$$

where $x_{i}$ represents the i  th sample, $c_{i}$ is the cluster to which $x_{i}$ belongs, $\mu_{ci}$ represents the center point corresponding to the cluster, and *M* is the total number of samples.

**Figure 6:**
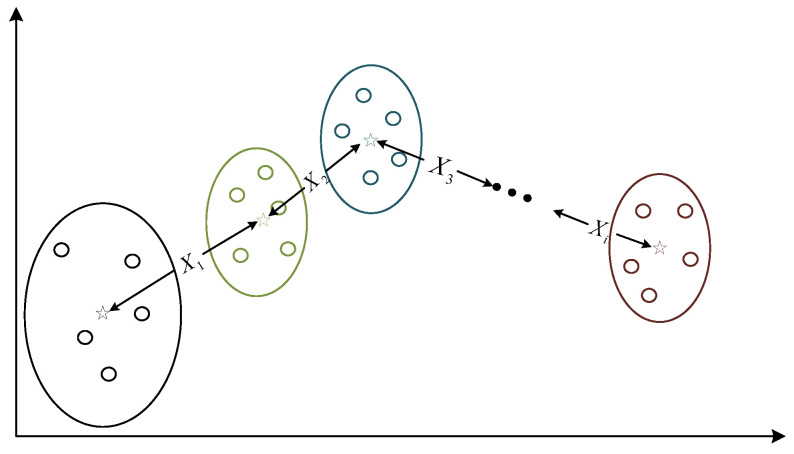
Schematic diagram of dynamic centroid algorithm.

As shown in Figure 6, first, we randomly select a sample point as the initial centroid, and then calculate the similarity between each sample point and the centroid. We classify each sample point into its most similar category, then recalculate the centroid point (i.e., the class center) of each class again, repeat this process until the centroid point no longer changes, and finally, the class and the class centroid can be obtained. We then divide the entire data cluster into a given number of K classes, but this class, divided according to data characteristics, may not have the trend we want, the size of each class may not be the same, and its data points may not be the same or continuous. We refer to this idea when calculating the migration trend of the feature distance, divide the data into n segments, find the centroid point for each segment, divide the entire data segment into n sample clusters, and in each sample cluster, the fluctuation distance between the data point and the centroid is calculated. The sum of the movement distance of the centroid and the fluctuation distance is the migration distance we want.

Therefore, here we divide the nonlinear fusion feature fea_kjade(*x_i_*,*y_i_*,*z_i_*), extracted from the feature fusion in the previous step, into n sample clusters, find the centroid *C*_0_ of the fault-free sample cluster, find the centroid point for each sample cluster separately *C_i_*, find the migration distance *X_i_* between the two centroid points, and find the fluctuation distance *d_i_* between the sample cluster and the previous centroid point *C_i_*_−1_. At this time, the required performance degradation evaluation index *ddd*_1_ can be obtained by adding these two distances.

The basic idea is to select the early fault-free samples initially judged in the KJADE fusion feature index as the fault-free sample clusters, and then divide the remaining samples into sample clusters according to the predetermined number of samples, calculate the centroids of the sample clusters, and specify each sample. The distance to its corresponding sample cluster centroid plus the migration distance from the sample cluster centroid point to the non-faulty sample cluster centroid point is the dynamic centroid distance of the sample. The pseudocode of the dynamic centroid algorithm is shown in Algorithm 1.
**Algorithm 1** Algorithm name: Dynamic centroid algorithm**Input:** KJADE feature feakjade after input wavelet packet decomposition and reconstruction.
1: *n*; Number of sample clusters.
2: *N*;The number of samples contained in each sample cluster.
3: [*idx, C*_0_] = *kmeans*(*fea_k_jade*(:, 1:350)′, 1); Find the coordinates of the no-fault centroid point.
4: **for** *i* = 1, 2, …*n* **do**
5: [*idx, C*_1_] = *kmeans*(*fea_k_jade*(:, 351 + *N* ∗ (*i* − 1):350 + *N* ∗ *i*)′, 1); Find the coordinates of the segmental centroid point.
6: **end for**
7: *d*(1, :) = *pdist*2(*C*_0_, *C*(1, :), ′*euclidean*′); Find the distance between the first two centroids
8: **for** *i* = 1, 2, …*n* − 1 **do**
9:   *d*(*i* + 1, :) = *pdist*2(*C*(*i*, :), *C*(*i* + 1, :), ′*euclidean*′); Find the coordinates of the two centroid points.
10: **end for**
11: *d*_3_(1, 1:350) = *pdist*2(*C*0*, feakjade*(:, 1:350)′, ′*euclidean*′); Find the migration distance between the fault-free data segment and the initial centroid point.
12: **for** *i* = 2, 3, …*n* − 1 **do**
13:  *d*_1_ = *pdist*2(*C*(*i*, :), *feakjade*(:, 351 + *N* ∗ (*i − *2):350 + *N* ∗ (*i* − 1))′, ′*euclidean*′); Find the fluctuation distance of the data.
14:  **for** *j* = 1, 2, …*i* **do**
15:  *d*_2_ = *sum*(*d*(1:*j*, :)); Calculate the sum of the distance of the centroid.
16:  **end for**
17:  *d*_3_(1, 351 + *N* ∗ (*i −* 2):350 + *N* ∗ (*i* − 1)) = *d*1 + *d*2; Calculate the total distance.
18:  **end for**
19:  *v*1 = *medf ilt*1(*d*_3_, 5); Median filter
20:  *ddd*1 = *mapminmax*(*v*1, 0, 1); Normalized
**Output:** Performance degradation indicator graph ddd1

## 3. Results and Discussion

**Figure 7:**
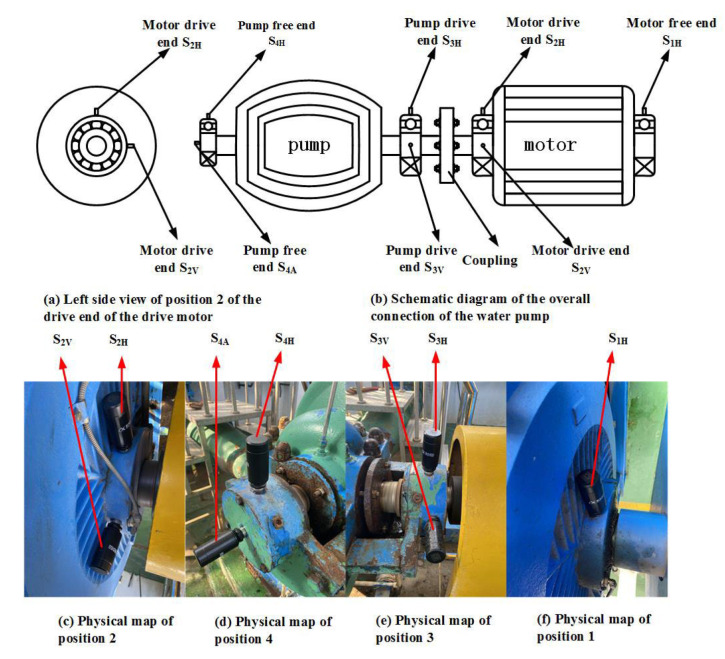
Water pump set equipment structure and vibration sensor installation physical diagram. (**a**) Left side view of position 2 of the drive end of the drive motor; (**b**) Schematic diagram of the overall connection of the water pump; (**c**) Physical map of position 2; (**d**) Physical map of position 4; (**e**) Physical map of position 3; (**f**) Physical map of position 1.

This paper takes the vibration signals collected by each measuring point of a pump from 11 September to 24 December 2021 as the analysis object. The physical diagram of the equipment structure of the pump set and the installation of the vibration sensor is shown in Figure 7. Vibration sensors in horizontal and vertical directions are placed on the free end and the driving end of the water pump and drive motor. The measuring point data sets are 1H, 2H, 2V, 3H, 3V, 4H, and 4A. Each half of each measuring point collects group data, eliminates the useless data due to problems such as shutdown and acquisition terminal failure during this period, and obtains 3684 groups of vibration signals during this period to complete the data preprocessing. The technical parameters related to the water pump are shown in Table 2, and the overall structure and physical diagram are shown in Figure 7.

### 3.1. Raw Signal Analysis

**Figure 8:**
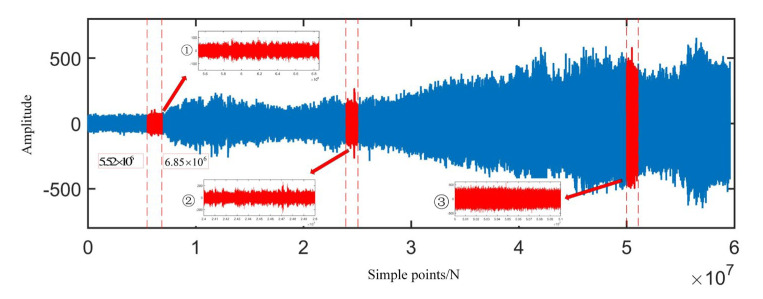
Time domain diagram of the original signal.

**Figure 9:**
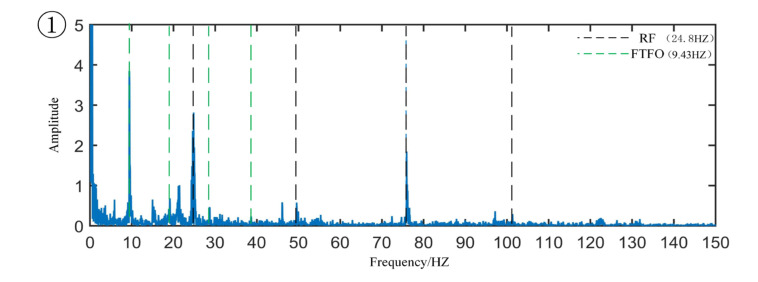
Minor fault signal envelope spectrum.

**Figure 10:**
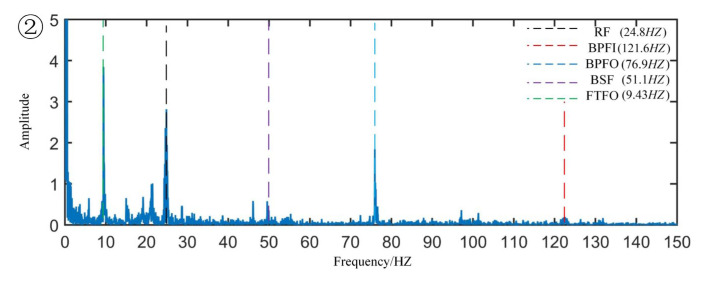
Mid-term fault signal envelope spectrum.

Figure 8 is the original signal time-domain diagram and partially enlarged diagram. From the partial magnification of the time domain signal, we can see the minor fault segment, that is, the data segment whose amplitude has not yet increased but the periodic shock component begins to appear. The coordinates of the corresponding sample points are 5,521,700 to 6,856,630. The envelope spectrum of the minor fault signal segment is shown in Figure 9. From the figure, one can observe the obvious cage fault frequency and rotation frequency and its frequency multiplication component. Regarding the sample of the mid-term fault section, the coordinates of the corresponding sample point are 24,000,000 to 25,000,000, and the envelope spectrum is drawn as shown in Figure 10, in which the outer raceway failure frequency, inner raceway failure frequency, rolling element failure frequency, and cage failure can be observed. This shows that in the severe fault section, the bearing fault evolved from the cage fault to a composite fault composed of the outer raceway fault, the inner raceway fault, the rolling element fault, and the cage fault. Regarding the samples of the later fault section, the coordinates of the corresponding sample points are 50,000,000 to 51,000,000, the main frequency component in the envelope spectrum is the rotation frequency, and there are sidebands around the rotation frequency, indicating that modulation occurs in the severe fault section.

**Figure 11:**
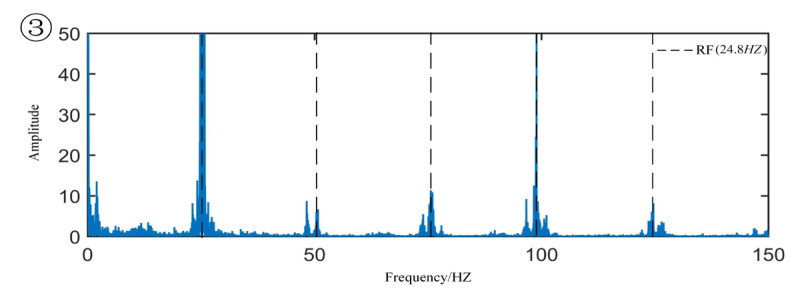
Later fault signal envelope spectrum.

For the minor fault segment, according to the number of sample points *n* = 16,384, the kurtosis value of the segment is calculated to draw the kurtosis curve of the original signal, as shown in Figure 11. The earliest increase in kurtosis occurs at sample segment 437, and the corresponding sample point coordinate is 7,159,808, indicating that a single kurtosis index has limitations in the early fault diagnosis of bearings and cannot accurately identify minor faults of bearings.

### 3.2. Analysis of Blind Separation and Wavelet Packet Reconstruction Processing Results

**Figure 12:**
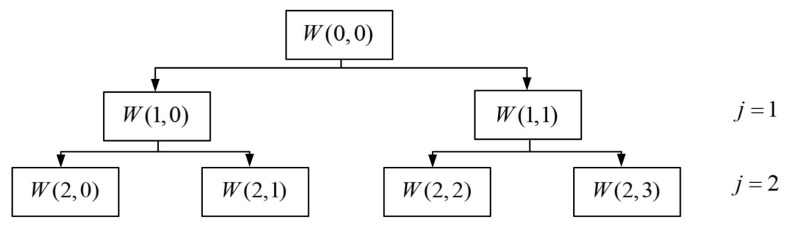
Wavelet decomposition tree.

In order to eliminate the interference of other vibration sources in the pump device to the signal acquisition under actual working conditions, the source signal is initially processed by the blind separation processing method, and wavelet packet decomposition is performed on the collected bearing vibration signal after blind separation. The decomposition structure is shown in Figure 12, where W(j, m) represents the node m of the layer j, each node represents the decomposition coefficient of the original signal *s*(*t*) on the scale j for the wavelet packet function, m represents the frequency band, and each node is associated with the corresponding frequency band match. In the figure, W(0, 0) represents the original signal, and other nodes such as W(2, 0) represent the 0th node coefficient of the second layer, and then each subband signal is extracted by reconstruction.

When the number of nodes m is an even number, it represents the low-frequency component signal decomposed by the low-pass filter coefficient h(k). On the contrary, when m is an odd number, it represents the high-frequency component signal decomposed by the high-pass filter coefficient g(k).

The sampling frequency of the bearing vibration signal is 12,800 Hz. The wavelet packet is decomposed into two layers, while the third layer has 2 ^ 2 = 4 frequency segments, and the frequency range of each frequency segment is 6400/4 = 1600 Hz. The specific range is shown in Table 3.

It can be seen from the table that the frequency bands corresponding to different nodes are different. Therefore, we analyze the spectrum of the signal after blind separation, create its spectrogram, observe the concentrated segment of the fault frequency, and select the wavelet node for the next step.

**Figure 13:**
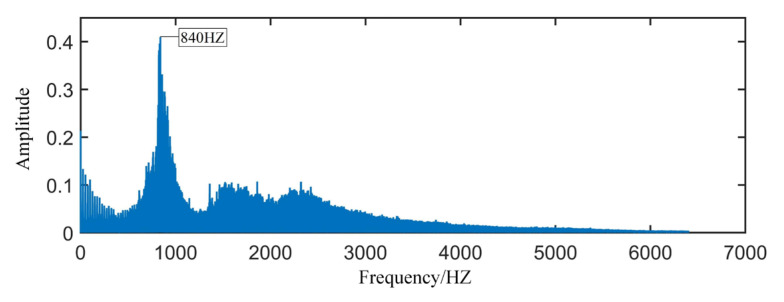
Spectrum of the signal after blind separation.

The bearing signal is a typical irregular signal. In order to grasp the time–frequency characteristics of each frequency band, the dbN wavelet can be used for multi-layer decomposition, and the time–frequency analysis of the signal is carried out in different frequency ranges. Because the db6 wavelet is a tightly supported orthogonal real wavelet, with good regularity and a large vanishing moment, it is used as the wavelet basis for wavelet packet decomposition. The second-order wavelet packet decomposition is performed on the signal after blind separation, and the db6 wavelet is selected as the wavelet base. Figure 13 is the spectrogram of the signal after blind separation processing. Through spectrogram analysis, the main frequency component of the signal is approximately 840 HZ. Therefore, node 4 (frequency band 1–1600 HZ) in the tree node is selected for reconstruction.

**Figure 14:**
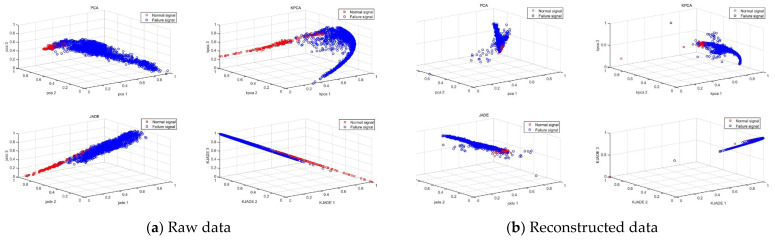
Feature point clustering.

To better verify the advantages of reconstructed signals, PCA, KPCA, JADE, and KJADE methods are used to fuse the original data and the reconstructed data, and the processed feature points are normalized and the first four are preliminarily selected. The normal working data of the bearing is used as a no-fault signal. Figure 14 shows the feature point clustering effect after feature fusion of source data and reconstructed data. Table 4 shows the intra-class distance of KJADE and JADE. By calculating the intra-class and inter-class distances between different feature points, it is found that the intra-class inter-class distances of the non-faulty samples and early fault samples in the reconstructed signal are significantly lower than the original signal, indicating that after blind separation and wavelet packet reconstruction, the clustering effect of feature points obtained by feature fusion of the four methods, PCA, KPCA, JADE, and KJADE, has been significantly improved.

### 3.3. Fusion Feature Distance Metrics

Considering that the three-dimensional coordinates of the signal feature points can only reflect the distribution and clustering effect of the feature points in the three-dimensional space, it cannot intuitively reflect the time node when the bearing starts to fail, and the trend of the subsequent bearing failure severity changes with time. The normal working data of the bearing in the first four days is used as the no-fault sample cluster, the centroid coordinates of the no-fault sample cluster are obtained, the distance between each subsequent feature point and the centroid of the no-fault sample cluster is calculated, and the distance index is used as the judgment bearing. A new indicator of the failure level is obtained.

To demonstrate the superiority of KJADE in the distance trend after the fusion of bearing fault features, feature distance calculation processing was performed on the data after feature fusion using PCA, KPCA, JADE, and KJADE methods. In terms of calculation, six different distance calculation methods, such as the Euclidean distance, Manhattan distance, and correlation distance, are used for verification.

**Figure 15:**
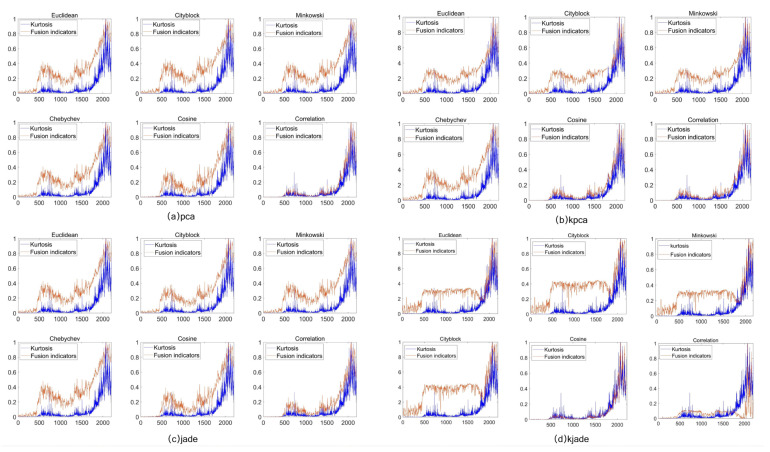
The effect of the comparison between the distance index and the cliff index without the distance calculation method. (**a**) PCA; (**b**) KPCA; (**c**) JADE; (**d**) KJADE.

Figure 15 is a trend diagram of the fusion feature distance indicators of the four feature fusion indicators PCA, KPCA, JADE, and KJADE under different distance calculation methods. The PCA fusion feature distance index has a large amplitude at the non-fault sample cluster, which cannot accurately judge the fault occurrence time. The angle cosine distance index and correlation distance index of the KPCA fusion feature have low amplitudes at the early non-fault sample clusters, which are in line with the actual working conditions, but the fault occurrence point is consistent with the kurtosis index, and early faults cannot be diagnosed in advance. There are also cases where the amplitudes of the remaining distance indicators are too large at the fault-free samples. The overall trend of JADE fusion features fluctuates greatly, and the amplitude is large at the fault-free sample cluster, which will interfere with the judgment of bearing faults. The Chebyshev distance index, the included angle cosine distance index, the Euclidean distance index, the Manhattan distance index, and the Minkowski distance index of the KJADE fusion feature also have the same problems as the above three other feature fusion methods. The amplitude at the cluster is abnormal, and the early warning of the early bearing failure cannot be realized.

**Figure 16:**
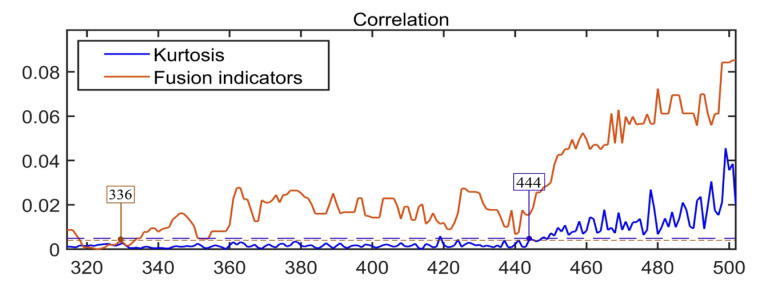
Local enlargement of the correlation distance index of the KJADE fusion feature.

The relevant distance index of KJADE effectively realizes the early warning of the early failure of the faulty bearing. Figure 16 shows a partially enlarged view of the correlation distance index of the KJADE fusion feature. The correlation distance index of the KJADE fusion feature rises at sample segment 336, and the corresponding sample coordinate is 5,505,024, which is the same as the starting sample coordinate of the minor fault segment in Section 3.1. 5,521,700 is basically the same, indicating that the correlation distance index of the KJADE fusion feature realizes the early diagnosis of minor bearing faults.

### 3.4. Cluster Migration Distance

In Section 3.2, after the early failure occurred, the kurtosis index and the KJADE fusion index showed a trend of first rising and then decreasing, and this anomalous phenomenon did not match the actual situation. In order to more accurately describe the trend of bearing failure degree with time, based on KJADE feature fusion, this paper proposes a novel performance degradation metric extraction method called Cluster Migration Distance (CMD). On the premise that the sample points corresponding to minor faults are determined in Section 3.2, all non-fault sample points are selected to obtain their centroids, divide the remaining sample points into n sample clusters according to the specified sample cluster capacity N, calculate the centroid of each sample cluster, and calculate the distance $X_{i}\left(i=1,2,3,\ldots ,N\right)$. In the subsequent calculation of the distance index of the sample points, the dynamic centroid method is used, that is, the distance $x_{j}\left(j=1,2,3\ldots \right)$ from each sample point to the centroid of the sample cluster where the sample point is located. The migration distance of the centroid point of the sample cluster is used as the dynamic centroid distance $v_{j}\;$ of the sample point. The specific calculation formula is as follows:

(27) $$v_{j}=\sum_{1}^{\left[j/n\right]+1}X_{i}+x_{j}$$

**Figure 17:**
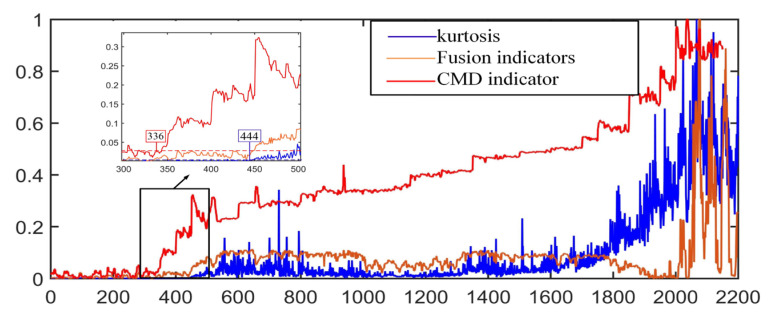
Dynamic center-of-mass metrics.

Considering the calculation accuracy and the total number of samples, this paper takes the sample cluster capacity *n* = 50 and the number of sample clusters as 37, calculates the dynamic centroid distance of each sample point, and draws its change trend as shown in Figure 17. Compared with the KJADE integrated index and the kurtosis index, the dynamic centroid distance index has a larger minor fault amplitude, which allows it to make a more accurate judgment on the minor fault of the bearing, and the subsequent amplitude shows an upward trend, which is more in line with actual engineering conditions.

In the early stages of equipment operation, the equipment operates normally, and various vibration indicators fluctuate normally. The fluctuations originate from changes in the flow and load of the pump. The bearing at the drive end of the pump has early normal wear characteristics, but there is no deterioration trend. With the operation of the pump, the corresponding fault information of the pump vibration data, such as kurtosis and other indicators, change significantly compared to the normal operation stage. At this time, the bearing will result in early failure. As the faulty bearing continues to work, various indicators fluctuate at high and low points. Intensified through the analysis of the data, the time-domain waveform can be seen in the time-domain waveform with insignificant swarm impact characteristics and the appearance of impact instability, indicating that the bearing is worn at this time, but the damaged surface of the rolling element is small, and the load area occasionally makes contact during the rotation process. When the inner and outer raceways are in contact, the vibration acceleration index rises, and when it is not in contact, the indicators decline.

As the bearing continues to work, the high-frequency vibration kurtosis density and impact energy ratio of the pump drive end change again compared to the previous ones. All kinds of indicators show a significant upward trend, and the vibration model shows new changes. The impact of the quasi-rotation frequency interval can be seen in the high-frequency vibration acceleration waveform, which appears to be stable, and the acceleration envelope spectrum is dominated by the rotation frequency and the fault frequency of the bearing inner ring, indicating that the main damage of the bearing at this stage is on the bearing inner ring. At the same time, the rising indicators show that the bearing damage surface is gradually expanding. The rise in vibration energy mainly comes from the bearing fault frequencies and sidebands in the frequency spectrum. The vibration velocity trend of the driving end and the free end of the pump shows that the effective value of the vibration velocity at the free end also shows a slow upward trend with time. This feature indicates that the vibration generated by the damage of the bearing at the driving end affects the entire shaft system of the pump.

It can be seen from Figure 17 that when the water pump is in a fault-free state, the distance between the fusion feature and the non-fault data center of mass is small. When the bearing is in a fault state, there will be a sudden change in the distance between the fusion coordinates and the non-fault center of mass, which can be determined at the moment of the sudden change, when the bearing failed. Among different fusion methods, we can see that compared with other feature fusion methods, the KJADE method is better than other feature fusion methods. When observing the trend of the feature distance, its change trend is more obvious. After the mutation, it can be clearly reflected by the relevant characteristic trend, so the state of the bearing during operation can be sensed in advance through the corresponding characteristic distance change. When the characteristic distance begins to grow larger or even begins to mutate, we can judge accordingly whether the bearing has failed.

When the kurtosis index is simply used, and when the number of sample points is 444, the kurtosis index begins to show a clear upward trend. At this time, the corresponding bearing failure stage is the mid-term failure of the bearing, and the early failure of the bearing cannot be accurately identified. At this time, the water pump bearing is running in a faulty state. The CMD index we proposed began to show an upward trend when the number of sampling points was 336, which was 20 h earlier than the simple kurtosis index. Early warning can be achieved, and economic losses caused by potential safety hazards can be prevented in time. 

## 4. Conclusions

In practical engineering applications, the pump bearing signal is seriously disturbed by noise, and the fault evolution is complex and changeable. Traditional performance degradation indicators cannot describe the degradation trend in a timely and accurate manner. To solve this problem, this work proposes a novel CMD index extraction method. This method can effectively eliminate the interference of the coaxial vibration source and can accurately describe the degradation trend of the bearing. The analysis results of an actual engineering case show that its early warning ability and index monotonicity are better than the traditional kurtosis index.

However, there are still some future challenges: (1) Better denoising of the pump bearing signal and extracting effective degradation indicators to achieve earlier warning and more accurate fault evolution process characterization, which is still a future challenge; (2) improving the computational efficiency of the method to achieve real-time monitoring, which requires further research; (3) in the process of CMD calculation, some parameters need to be manually selected, and the method of realizing parameters’ self-optimization deserves further study; and lastly, (4) the automatic selection and fusion of features is also worthy of further research to improve the clustering of features.

## Figures and Tables

**Table 1:** Time and frequency domain and time–frequency domain characteristic indicators.

Features	Expression	Features	Expression
Mean	$F1=\frac{1}{N}\sum {}_{i=1}^{N}x_{i}(t)$	Center frequency	$F13=\sum {}_{i=1}^{N}f_{i}s_{i}/\sum {}_{j=1}^{N}s_{j}$
Root mean square value	$F2=\sqrt{\frac{1}{N}\sum {}_{i=1}^{N}x_{i}^{2}(t))}$	Frequency standard deviation	$F14=\frac{1}{N}\sum {}_{j=1}^{N}(s_{j}-\frac{1}{N}\sum {}_{i=1}^{N}s_{i})$
Square root magnitude	$F3={\left[\frac{1}{N}\sum {}_{i=1}^{N}\sqrt{\left|x_{i}(t)\right|}\right]}^{2}$	Root mean square frequency	$F15=\sqrt{\sum {}_{i=1}^{N}f_{i}^{2}s_{i}/\sum {}_{j=1}^{N}s_{j}}$
Absolute mean	$F4=\frac{1}{N}\sum {}_{i=1}^{N}\left|x_{i}(t)\right|$	First Band	$F17=E_{0}/E$
Kurtosis	$F5=\frac{1}{N}\sum {}_{i=1}^{N}x_{i}^{4}(t)$	Second Band	$F18=E_{1}/E$
Variance	$F6=\frac{1}{N-1}\sum {}_{i=1}^{N}{\left(x_{i}-F1\right)}^{2}$	Third Band	$F19=E_{2}/E$
Waveform Indicator	$F7=\frac{F2}{F4}$	Fourth Band	$F20=E_{3}/E$
Peak indicator	$F8=\frac{\max(x_{i}(t))}{F2}$	Fifth Band	$F21=E_{4}/E$
Impulse indicator	$F9=\frac{\max(x_{i}(t))}{F4}$	Sixth Band	$F22=E_{5}/E$
Margin indicator	$F10=\frac{\max(x_{i}(t))}{F3}$	Seventh Band	$F23=E_{6}/E$
Skewness Indicator	$F11=\frac{\frac{1}{N}\sum {}_{i=1}^{N}x_{i}^{3}(t)}{F2^{3}}$	Eighth Band	$F24=E_{7}/E$
Kurtosis indicator	$F12=\frac{F5}{F2^{4}}$		
Absolute mean	$F16=\frac{1}{N}\sum {}_{i=1}^{N}[s_{i}{\left(f_{i}-F13\right)}^{4}]/{\left(\frac{1}{N}\sum_{j=1}^{N}\left(s_{j}{\left(f_{i}-F_{13}\right)}^{2}\right)\right)}^{2}$		

**Table 2:** Pump-related technical parameters.

Items	Parameters	Items	Parameters
power	220 Kw	COS *∅*	0.85
frequency	50 HZ	work schedule	S1
Phase	3	Insulation class	155
Voltage	6000 V	Rotating speed	1490 r/min
current	26.8 A	cooling method	IC611
Rotating speed	1480 r/min	Diameter of impeller	448 mm
Equipped with power	220 KW	flow	720 m^3^/h
Rotating speed	1490 RPM	frequency conversion	24.8 HZ
BPFI	121.6 HZ	BPFO	76.9 HZ
BSF	51.6 HZ	FTFO	9.43 HZ

**Table 3:** Frequency range.

Node	W(2,0)	W(2,1)	W(2,2)	W(2,3)
Frequency range/HZ	0~1600	1601~3200	3201~4800	4801~6400

**Table 4:** Intra-class distance.

	Intra-Class Distance of KJADE	Intra-Class Distance of JADE
Raw data	0.7606	1.4764
reconstruct data	0.0776	0.8045
	**Intra-Class Distance of KPCA**	**Intra-Class Distance of PCA**
Raw data	1.043	0.9804
reconstruct data	0.8771	0.7566

## Data Availability

Not applicable.
